# Supplementary material for: Phase transition kinetics of superionic H2O ice phases revealed by Megahertz X-ray free-electron laser-heating experiments
Source: Nat Commun. 2024 Sep 23;15:8256. doi: 10.1038/s41467-024-52505-0 (PMC11420352; doi:10.1038/s41467-024-52505-0)
Supplement: Supplementary file 1 — Supplementary Information [file 41467_2024_52505_MOESM1_ESM.pdf]

Supplementary Information: Phase transition kinetics of superionic H_2O ice phases revealed by Megahertz X-ray free-electron laser-heating experiments

R. J. Husband[1*], H. P. Liermann[1], J. D. McHardy[2], R. S. McWilliams[2], A. F. Goncharov[3], V. B. Prakapenka[4], E. Edmund[3], S. Chariton[4], Z. Konôpková[5], C. Strohm[1], C. Sanchez-Valle[6], M. Frost[7], L. Andriambariarijaona[8], K. Appel[5], C. Baehtz[9], O. B. Ball[2], R. Briggs[10], J. Buchen[11,a], V. Cerantola[5,b], J. Choi[12], A. L. Coleman[10], H. Cynn[10], A. Dwivedi[5], H. Graafsma[1], H. Hwang[1,c], E. Koemets[11,d], T. Laurus[1], Y. Lee[12], X. Li[1,e], H. Marquardt[11], A. Mondal[6], M. Nakatsutsumi[5], S. Ninet[8], E. Pace[2], C. Pepin[13], C. Prescher[14], S. Stern[1,f], J. Sztuk-Dambietz[5], U. Zastrau[5], and M. I. McMahon[2*]

[1]Deutsches Elektronen-Synchrotron DESY, Notkestr. 85, 22607 Hamburg, Germany

[2]SUPA, School of Physics and Astronomy, and Centre for Science at Extreme Conditions, The University of Edinburgh, Peter Guthrie Tait Road, Edinburgh EH9 3FD, United Kingdom

[3]Carnegie Science, Earth and Planets Laboratory, 5241 Broad Branch Road, NW, Washington, DC 20015, USA

[4]The University of Chicago, Center for Advanced Radiation Sources, 5640 South Ellis Avenue Chicago, IL 60637, USA

[5]European XFEL, Holzkoppel 4, 22869 Schenefeld, Germany

[6]Universität Münster, Institut für Mineralogie, Corrensstraße 24, 48149 Münster, Germany

[7]SLAC National Accelerator Laboratory, 2575 Sand Hill Road, California 94025, USA

[8]Institut de Minéralogie, de Physique des Matériaux et de Cosmochimie (IMPMC), Sorbonne Université, CNRS UMR 7590, MNHN, 4, place Jussieu, Paris, France

[9]Institute of Radiation Physics, Helmholtz-Zentrum Dresden-Rossendorf, Bautzner Landstraße 400, 01328 Dresden, Germany

[10]Lawrence Livermore National Laboratory, Livermore, California 94550, USA

[11]Department of Earth Sciences, University of Oxford, South Parks Road, Oxford OX1 3AN, United Kingdom

[12]Department of Earth System Sciences, Yonsei University, Seoul 03722, Korea

[13]CEA, DAM, DIF, 91297 Arpajon, France; Université Paris-Saclay, CEA, Laboratoire Matière en Conditions Extrêmes, 91680 Bruyères-le-Châtel, France

[14]Institute of Earth and Environmental Sciences, University of Freiburg, Freiburg, Germany.

[a]Current address: Bayerisches Geoinstitut, Universität Bayreuth, Universitätsstraße 30, 95447 Bayreuth, Germany

[b]Current address: Department of Earth and Environmental Sciences (DISAT), University of Milano-Bicocca, Piazza della Scienza 4, 20126 Milan, Italy.

[c]Current address: School of Earth Sciences and Environmental Engineering, Gwangju Institute of Science and Technology, 123 Cheomdan-Gwagiro, Gwangju 61005, Republic of Korea.

[d]Current address: Diamond Light Source Ltd, Harwell Science and Innovation Campus, Didcot, Oxfordshire, OX11 0DE, UK

[e]Current address: Synergetic Extreme Condition High-Pressure Science Center, State Key Laboratory of Superhard Materials, College of Physics. Jilin University 130012, China

[f]Current address: X-Spectrum GmbH, Luruper Hauptstraße 1, 22547 Hamburg, Germany

*Corresponding authors. Email: rachel.husband@desy.de, M.I.McMahon@ed.ac.uk.

Supplementary Table 1: Summary of runs in which SI-bcc ice was observed. Runs in which SI-fcc ice was observed are in bold. The X-ray pulse energy given corresponds to the average pulse energy across all 300 pulses, and the standard deviation is given in parenthesis.

**DAC**	**Culet size (μm)**	**Sample**	**P (GPa)**	**Coupler**	**Beam size**	**Run number**	**X-ray Trans-mission (%)**	**X-ray pulse energy (μJ)**
DAC 1	250	H_2O	26.2	Fe_3O_4	26 μm	831, 832, 833, 835, 836, 837, 838, 839, 841, 843, 848, 849, 850, 851, 852, 853, 854	5, 7, 9, 13, 15, 17, 19, 21, 25, 29, 31, 33, 35, 37, 39, 39, 43	5.7(1.3), 5.3(1.3) 10.0 (1.3), 19(2) 22(3), 26(3) 31(4), 27(3) 38(5), 43(5) 42(5), 45(5) 45(5), 51(6) 46(6), 47(6) 64(6)
DAC 2	200	H_2O	27.0	Au	13 μm	342, 343	3, 3.5	4.6(1.1), 6.9(0.8)
DAC 3	300	H_2O	30.1	Cu	26 μm	874, 875, 876, 877, 878, 879	11.5, 15, 20, 25, 35, 45	20(3), 24(3) 31(4), 41(4) 54(7), 55(8)
DAC 4	300	H_2O	36.7	Ag	<8 μm	917: 10, 11, 12, 13, 14, 15, 16, 17, 18	26, 29, 32, 50, 60, 70, 80, 90, 100	35(4) 32(4), 33(4) 63(7), 74(8) 86(10), 95(15) 99(14), 113(12)
		H_2O	39.8	Au	<8 μm	918: 5, 6, 7	10, 12, 16	6.2(1.0), 13.9(1.7) 21(3)
DAC 5	300	H_2O+ He	38.4	Au	26 μm	804, 805, 807, 808, 809, 810, 812, 824	9, 11.5, 13, 15, 16, 17, 18, 43	10.0(1.6), 15(2) 13(3), 8(2) 11(2), 22(3) 20(3), 60(8)
DAC 6	200	H_2O	49.9	PtIr	13 μm	293, **296**, 297, 298	10, 15, 15, 12	10.5(1.6), 21(2) 22(3), 21(3)
DAC 7	300	H_2O	57.5	Rh	<8 μm	618	90	121(12)
DAC 8	200	H_2O	69.3	Ag	<8 μm	502, **504**, 505, **506**, **508**, 509, 510, 513, **568** (26 μm beam size)	25.5, 28, 29, 30, 32, 33, 34, 37, 50	41(5), 43(6) 47(5), 56(6) 42(5), 51(6) 48(5), 56(6) 100(10)
		H_2O	67.3	Cu	<8 μm	529, 532, 533, **534**, **535**, **536**	30, 60, 70, 80, 90, 100	39(5), 76(9) 116(10), 80(10) 133(14), 116(13)
		H_2O	64.1	Au	<8 μm	556, 558, 559, 560	21, 30, 32, 34	25(3), 33(5) 44(4), 38(5)
DAC 9	300	H_2O	34.2	Rh	13 μm	87	25	73(14)

Supplementary Table 2: Summary of results for all samples, which were determined from the runs given in Supplementary Table 1. For all samples apart from DAC 9, the *d*-spacing of the cold (room temperature) ice VII (110) reflection was determined from the integrated pattern from the first pulse and the *d*-spacing of the high temperature reflections were determined from the peak histogram. Peak histograms were not produced for DAC 9 because this sample showed evidence of contamination, and so the d-spacing of the high temperature reflection was determined from the integrated pattern of XRD images summed over pulses 51-300. Both bcc and fcc crystal structures were considered for phase identification, and the corresponding densities are given in the table for comparison. For peaks identified as SI-bcc, the density determined by the fcc indexing was too high for a high temperature state.

		**Ice VII (110) (pulse 1)**		**New, high temperature peak (pulses 51-300)**			
**DAC**	**Coupler**	***d*-spacing (Å)**	**Density (g/cc)**	***d*-spacing (Å)**	**Density assuming SI-bcc (110) (g/cc)**	**Density assuming SI-fcc (111) (g/cc)**	**Phase identification**
DAC 1	Fe_3O_4	2.090	2.317	2.150	2.129	2.318	SI-bcc
DAC 2	Au	2.086	2.330	2.139	2.162	2.353	SI-bcc
DAC 3	Cu	2.071	2.381	2.129	2.193	2.387	SI-bcc
DAC 4	Ag	2.043	2.481	2.091	2.314	2.519	SI-bcc
	Au	2.031	2.525	2.094	2.303	2.507	SI-bcc
DAC 5	Au	2.037	2.503	2.092	2.311	2.516	SI-bcc
DAC 6	PtIr	1.998	2.652	2.053	2.443	2.660	SI-bcc
	PtIr			2.109	2.255	2.455	SI-fcc
DAC 7	Rh	1.978	2.733	2.025	2.546	2.771	SI-bcc
DAC 8	Ag	1.950	2.853	2.010	2.603	2.834	SI-bcc
	Ag			2.068	2.391	2.617	SI-fcc
	Cu	1.955	2.831	2.006	2.619	2.851	SI-bcc
	Cu			2.065	2.404	2.603	SI-fcc
	Au	1.962	2.801	2.018	2.573	2.801	SI-bcc
DAC 9	Rh	2.052	2.446	2.098	2.291	2.494	SI-bcc

Supplementary Table 3: Summary of temperatures determined from SOP. The temperature in each 9.1 ns time window during the heating cycle was estimated based on a fit of a Planck function to thermal emission in the 600-775 nm spectral range, and averaged to determine the mean temperature of the run. The standard deviation is given in parenthesis. The tabulated mean temperatures are rounded to the first digit of the standard deviation. Thermal emission from some runs was too low for reliable temperature determination, which is indicated by n/a in the table. Data are also missing for other reasons; for example, the fit to the spectra from runs 297 and 824 was deemed to poor for temperature determination, and the streak camera data from run 536 was corrupted.

**DAC**	**Coupler**	**P (GPa)**	**Run number**	**Mean temperature (K)**
DAC 1	Fe_3O_4	26.2	831, 832, 833, 835, 836, 837, 838, 839, 841, 843, 848, 849, 850, 851, 852, 853, 854	n/a, n/a, n/a, n/a, n/a, n/a, n/a, n/a, n/a, n/a, n/a, n/a, n/a, n/a, n/a, n/a, n/a
DAC 2	Au	27.0	342, 343	n/a, 2100(300)
DAC 3	Cu	30.1	874, 875, 876, 877, 878, 879	n/a, n/a, n/a, n/a, n/a, 3200(500)
DAC 4	Ag	36.7	917: 10, 11, 12, 13, 14, 15, 16, 17, 18	n/a, n/a, n/a, 2400(600), 2400(300), 2400(600), 2300(400), 2300(300), 2300(400)
	Au	39.8	918: 5, 6, 7	n/a, n/a, 2700(500)
DAC 5	Au	38.4	804, 805, 807, 808, 809, 810, 812, 824	2600(300), 2600(400), 2300(200), n/a, 2100(200), 2400(300), 2200(200), n/a
DAC 6	PtIr	49.9	293, 296, 297, 298	1700(80), n/a, n/a, n/a
DAC 7	Rh	57.5	618	n/a
DAC 8	Ag	69.3	502, 504, 505, 506, 508, 509, 510, 513, 568 (26 µm beam size)	n/a, n/a, n/a, n/a, n/a, n/a, n/a, n/a, n/a
	Cu	67.3	529, 532, 533, 534, 535, 536	3000(700), 2700(500), 2500(400), 2600(500), 2300(500), n/a
	Au	64.1	556, 558, 559, 560	n/a, 2800 (800), 2700(500), 2600(200)
DAC 9	Rh	34.2	87	n/a

Supplementary Note 1

DAC preparation

Data were collected from a total of 9 symmetric diamond anvil cells (DACs) loaded with H_2O. Microscope images of the samples before the XFEL experiment are shown in Supplementary Fig. 1, which were collected after the sample was compressed to the final pressure for data collection. DACs 1-8 were prepared with Re gaskets, whereas DAC 9 was prepared with a W gasket. DACs 1 and 2 were prepared with nanopower couplers (Au and Fe_3O_4) which were dispersed throughout the sample chamber. A small piece of Au foil was included in the sample chamber of DAC 1 for pressure determination using X-ray diffraction (XRD). DACs 3, 5, 6, 7, and 9 were prepared with foil couplers containing an array of small holes (~6 - 22 µm diameter) which were cut using a laser drilling machine. In DAC 7, the Rh coupler was insulated from the diamond anvil by a layer of Al_2O_3. No insulating layer was used in DACs 3, 5, and 6, and the foil was assumed to be insulated from the diamond anvil by a thin layer of H_2O. DAC 9 was initially loaded with a thick Rh foil which completely filled the gasket hole, after which 5-6 µm of the foil was removed from each side using an electrode discharge machine (EDM) to provide an insulating layer of H_2O. DAC 5 was loaded with a H_2O-He mixture, which separated into two distinct regions during crystallization compression. All data shown here were collected from the H_2O region in the vicinity of the coupler.

DACs 4 and 8 were prepared with three different coupler materials embedded in the Re gasket using the design shown in Fig. 1. Three sample chambers were cut in the pre-indented (~17-18 µm thick) gasket using a laser drilling machine, and filled with Au, Cu and Ag foil. The same laser was used to cut a small (~15 µm diameter) hole in each of the foils to produce a doughnut-type coupler shape. An EDM was used to remove a ~4 µm thick surface layer on both sides of the gasket (darkened region on the microscope photo), which provided a thin H_2O layer to prevent direct contact of the gasket or coupler with the diamond anvil.

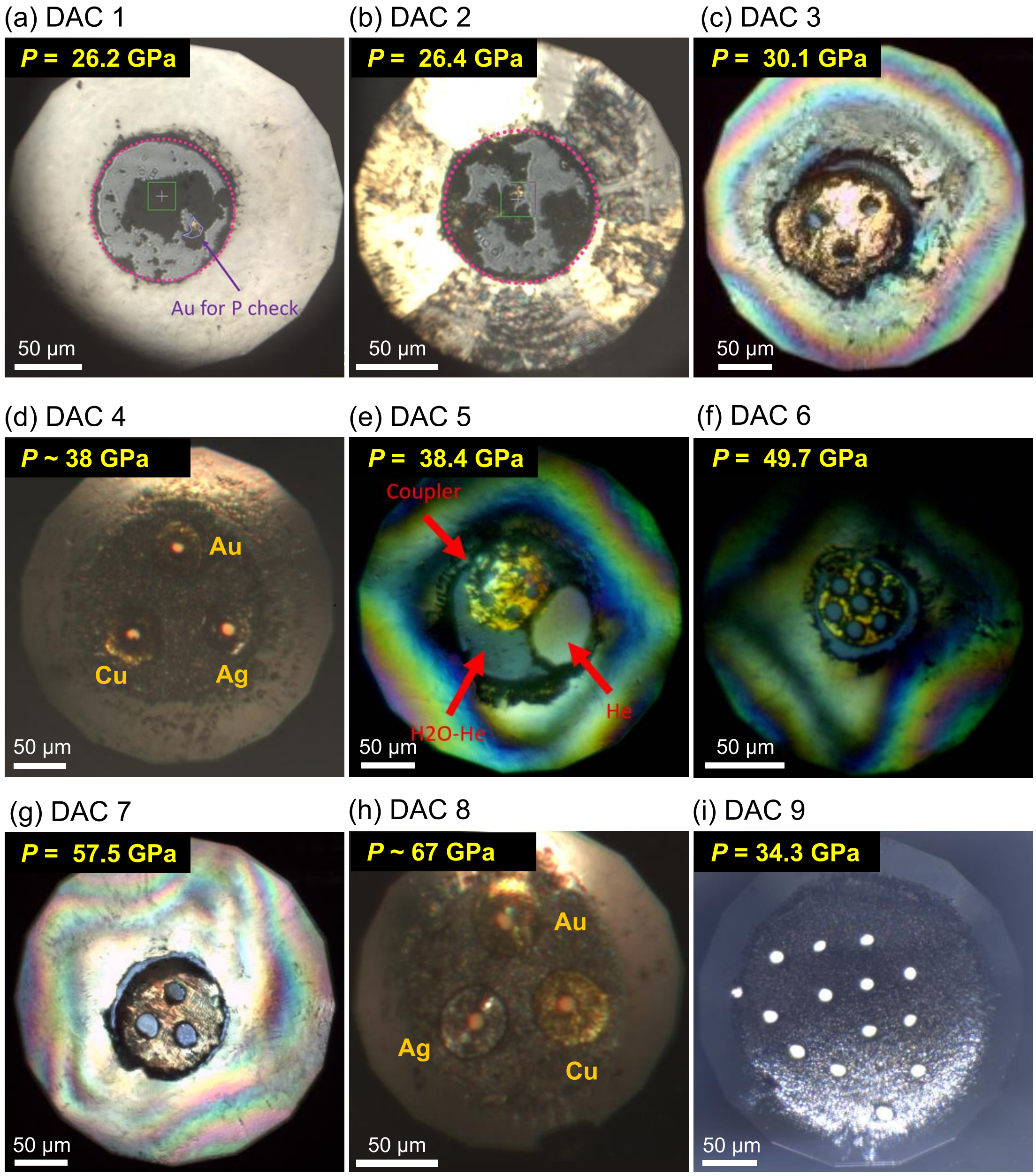

Supplementary Figure 1: Microscope images of samples before the XFEL experiment. Further details are given in Supplementary Table 1. Panels (c), (e), (f), and (g) were collected under polarized light. The pressure in DACs 4 and 5 was found to be different in each of the coupler holes due to a pressure gradient within the sample chamber; the average pressure is therefore indicated on these images. No data were collected from the Cu coupler in DAC 4. Scale bars are approximate, and were determined based on the expected culet dimensions of the diamond anvils.

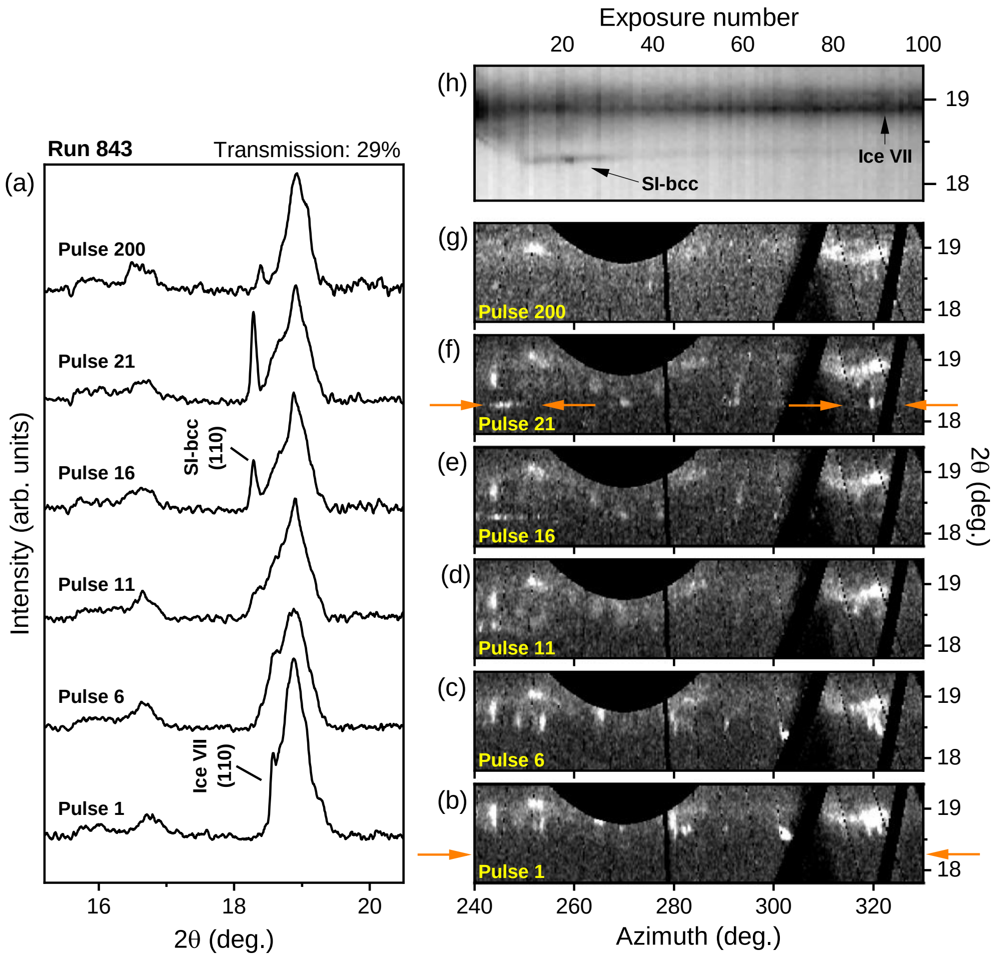

Supplementary Figure 2: XRD data collected at 26.2 GPa. These data were collected from DAC 1 using 29% transmission and a dispersed Fe_3O_4 nanopowder coupler (run 843). (a) Selected XRD patterns, (b)-(g) corresponding unwrapped (2θ-φ) XRD images, and (h) time-dependent changes in integrated patterns collected with the first 100 pulses in the run. Broadening of the ice VII (110) reflection in the first pulse pattern in (a) is indicative of the presence of non-hydrostatic stress. No significant change in the peak width is observed on heating; instead, the SI-bcc (110) reflection is observed at lower angles. The orange arrows in (f) indicate the two most intense SI-bcc diffraction spots in this image. No diffracted signal is observed at this 2θ value in XRD data collected from the cold sample, illustrated by the orange arrows in (b). Source data are provided as a Source Data file.

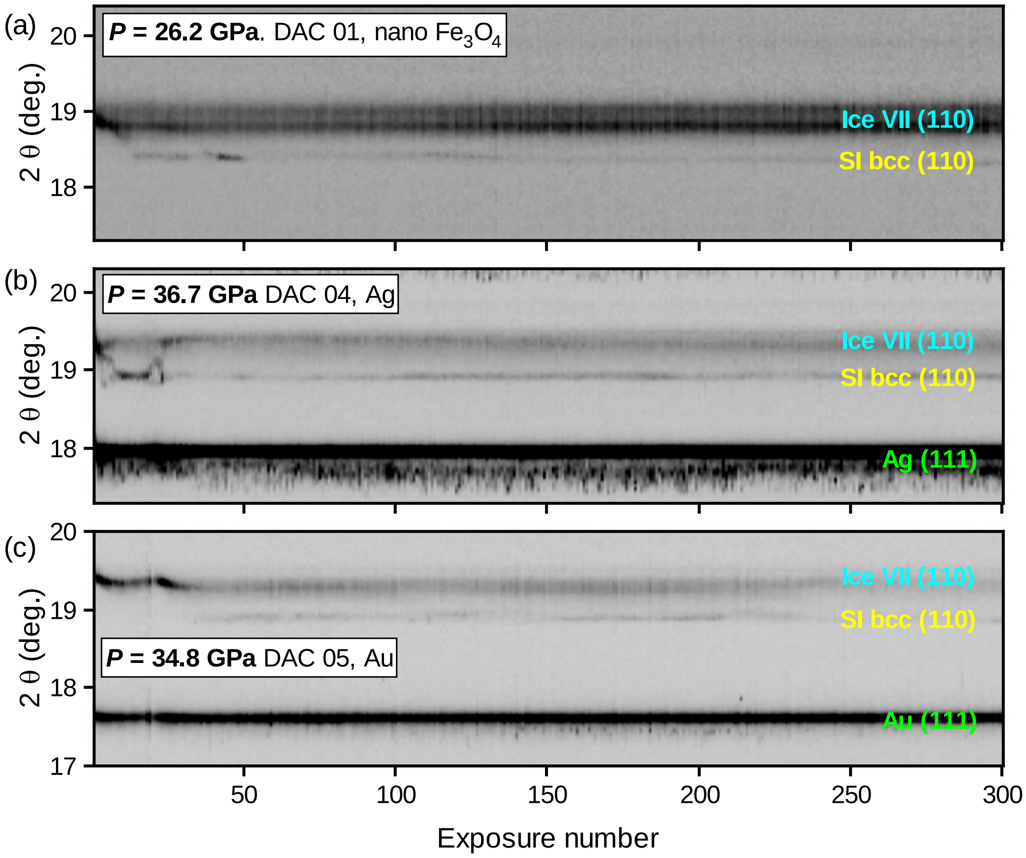

Supplementary Figure 3: Evolution of integrated XRD patterns for selected runs in which SI-bcc ice was observed. (a) XRD data from run 833, which was collected from DAC 1 (26.2 GPa) at 9% transmission. Nanopowder Fe_2O_3 was used as a coupler; however, the diffraction peaks were too weak to be observed. (b) XRD data from run 917, which was collected from DAC 4 (36.7 GPa) using 16% transmission. An embedded Ag doughnut coupler was used, and heating of Ag is clearly visible from the low angle contribution to the (111) reflection. (c) XRD data from run 809, which was collected from DAC 5 at 16% transmission. A small volume of He was present in the sample chamber, but this region was not targeted in this experiment. A hole in an Au foil was used as a coupler. Source data are provided as a Source Data file.

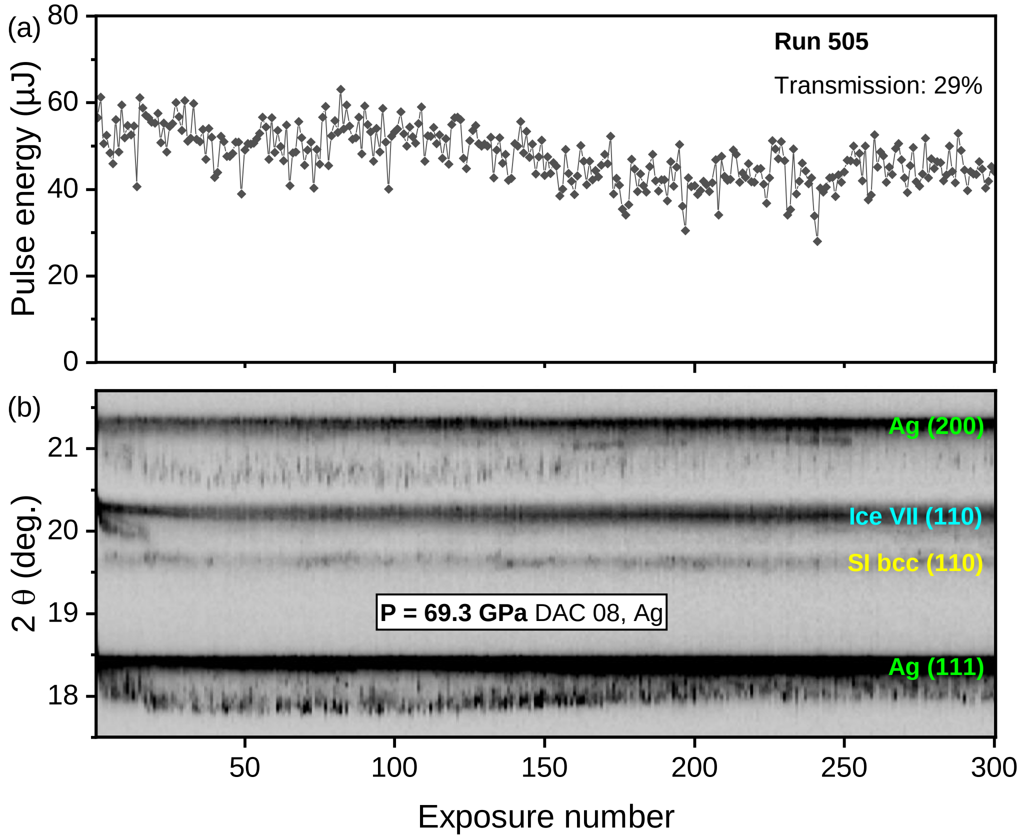

Supplementary Figure 4: Evolution of the pulse energy and integrated XRD patterns collected at 69.3 GPa. These data were collected with 29% X-ray transmission from the Ag doughnut coupler in DAC 8 (run 505). Data from this run are also shown in Fig. 3. (a) X-ray pulse energy incident on the sample, which was measured using the intensity and position monitor (IPM) situated upstream of the sample position. The IPM was calibrated using an upstream X-ray gas monitor (XGM) before the start of the experiment. Pulse energy values were not corrected to account for absorption by the upstream diamond anvil. (b) Integrated XRD patterns collected during the run, where small variations in diffracted signal from hot Ag are present due to variations in the pulse energy. The evolution of the SI-bcc (110) reflection, however, is relatively smooth. Source data are provided as a Source Data file.

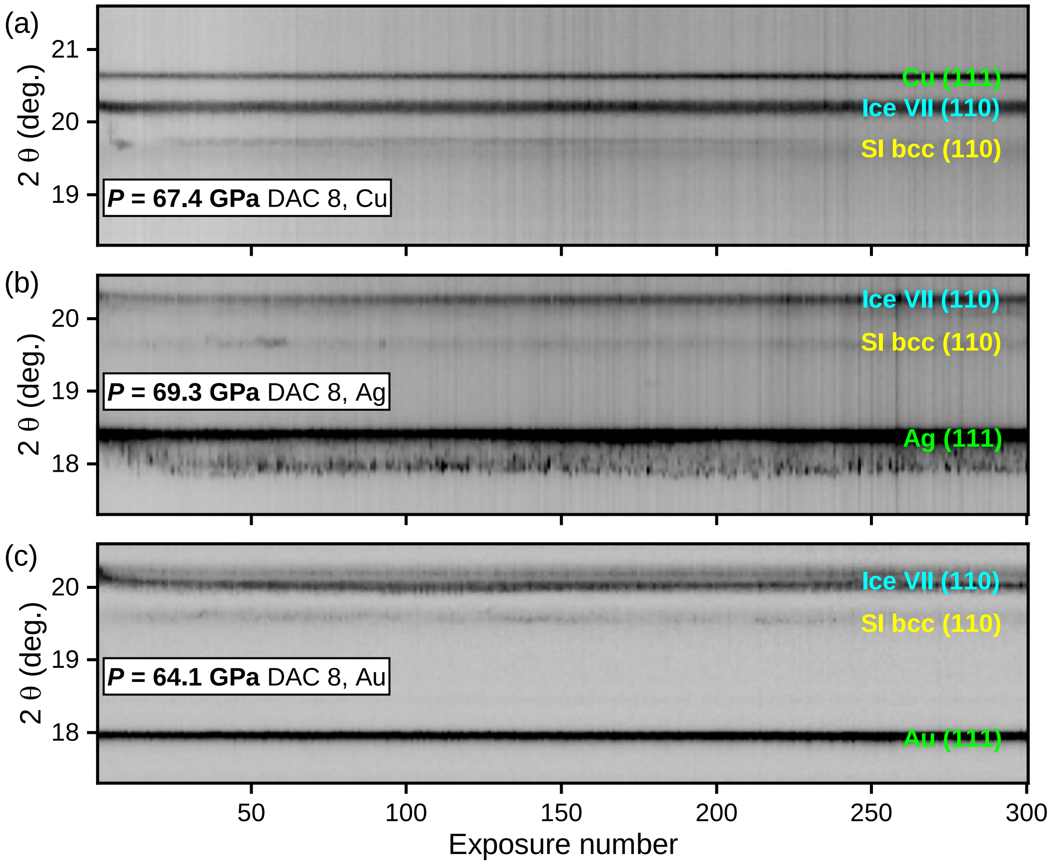

Supplementary Figure 5: Evolution of integrated XRD patterns in runs collected from embedded doughnut couplers in DAC 8. (a) XRD data from run 532, which was collected from the Cu coupler (67.4 GPa) at 60% transmission. Data from this run are also shown in Fig. 6. (b) XRD data from run 504, which was collected from the Ag coupler (36.7 GPa) using 28% transmission. (c) XRD data from run 560, which was collected from the Au coupler (64.1 GPa) at 16% transmission. SI-bcc is observed in all three runs, but no evidence of SI-fcc was observed. A strong signal from thermally-expanded Ag is observed in (b), which originates from the hot coupler, whereas the Cu and Au lines only show a very small thermal expansion which is not visible in the figures. Source data are provided as a Source Data file.

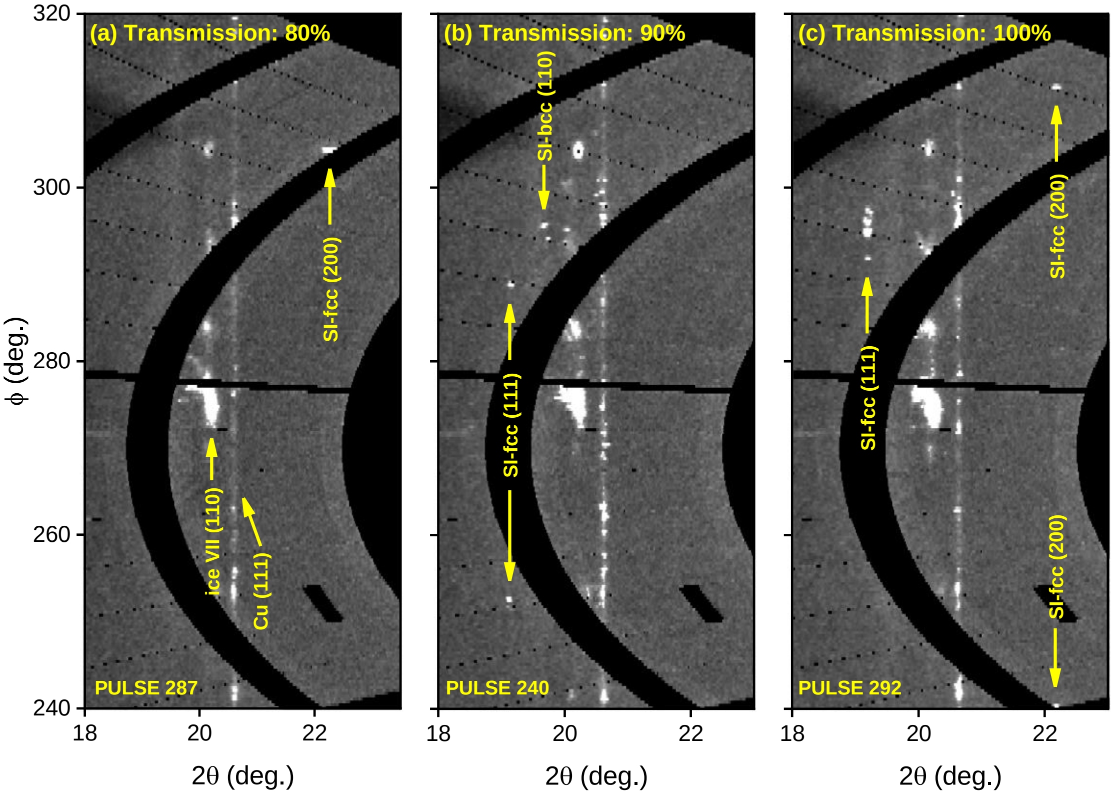

Supplementary Figure 6: **Observation of SI-fcc at 67.3 GPa.** Panels (a)-(c) show unwrapped (2θ-φ) XRD images collected from the embedded Cu doughnut coupler at DAC 8. The images correspond to: (a) pulse 287 in run 534, (b) pulse 240 in run 535, and (c) pulse 292 in run 536. The SI-fcc (200) reflection is observed in (a) and (c), whereas the SI-fcc (111) reflection is observed in (b) and (c). Source data are provided as a Source Data file.

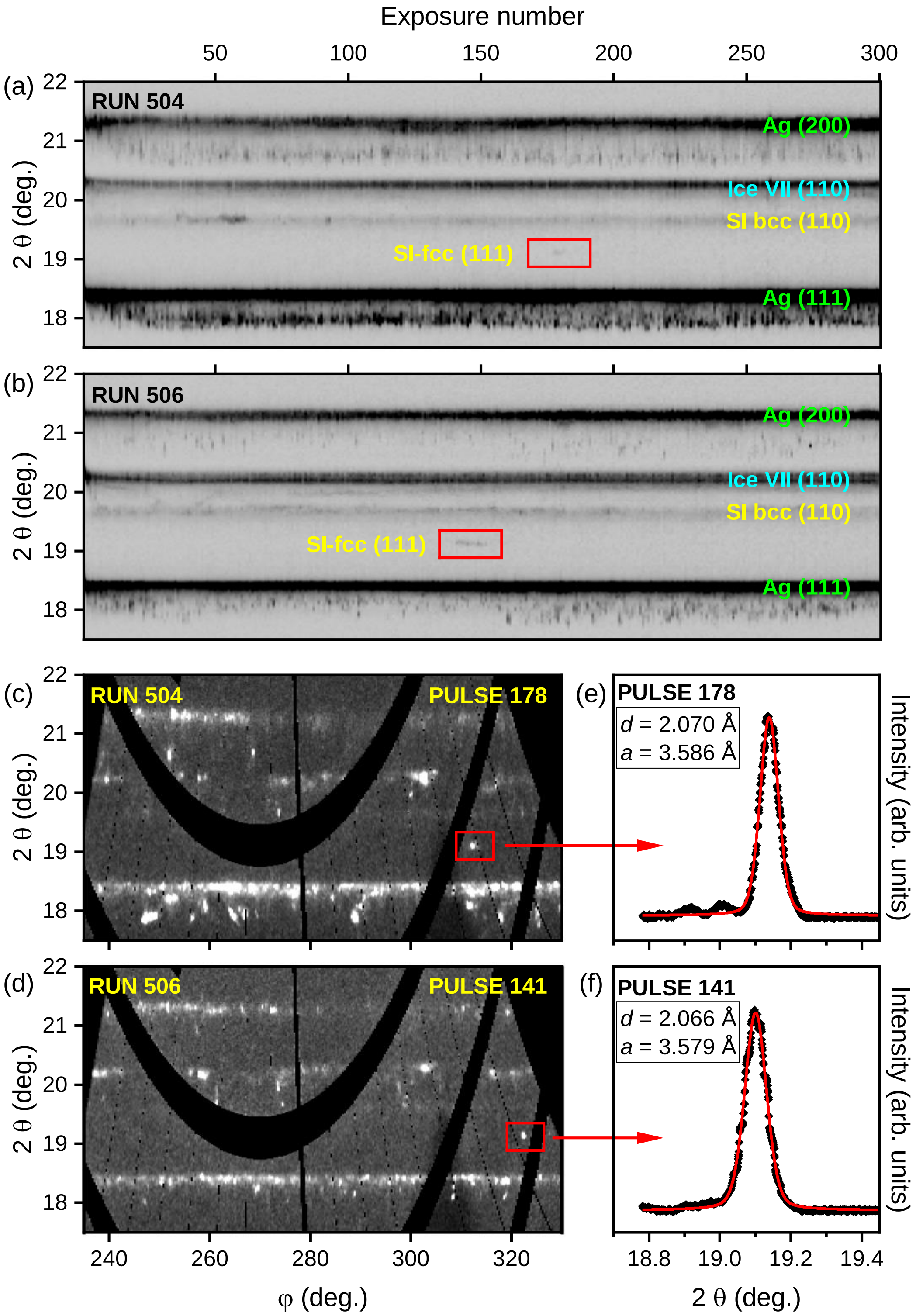

Supplementary Figure 7: Observation of SI-fcc at 69.3 GPa. Data were collected from the Ag doughnut coupler in DAC 8**.** (a) and (b) show the temporal evolution of the integrated XRD patterns in runs (a) 504 and (b) 506, respectively. The red rectangles indicate the regions where evidence of SI-fcc was observed during the run. In both cases, the SI-fcc signal comes from an individual diffraction spot, which is highlighted by the red rectangles in panels (c) and (d), which show unwrapped (2θ-φ) XRD images from runs 504 and 506, respectively. The integrated XRD pattern determined from an ROI around the SI-fcc diffraction spots in (c) and (d) are shown in panels (e) and (f), respectively. In (e) and (f), the black points show the experimental data and the red line shows a fit using a Pseudo-Voigt function, where the *d*-spacing and lattice parameter calculated from the refined peak position are given in the inset. Source data are provided as a Source Data file.

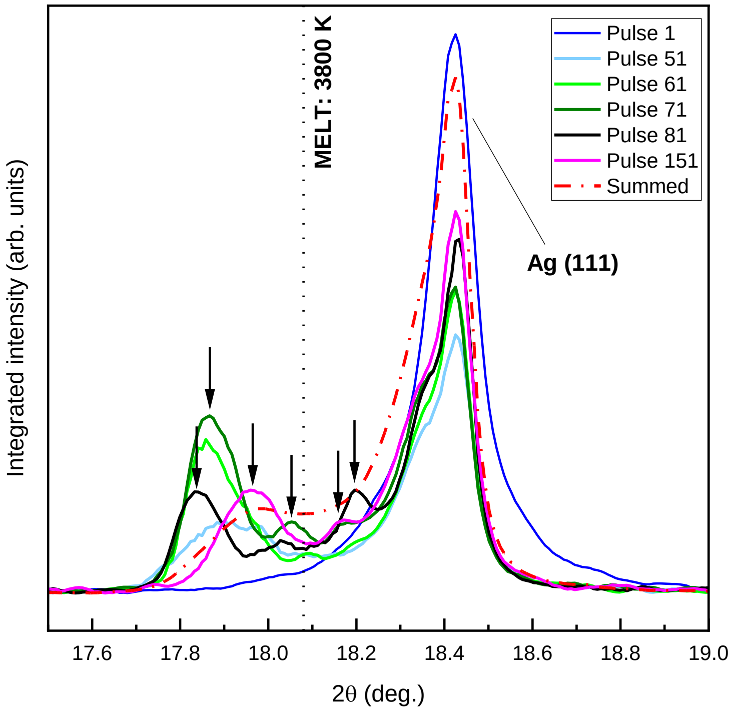

Supplementary Figure 8: Selected integrated XRD patterns collected at 69.3 GPa. These data were collected from the Ag embedded coupler in DAC 8 (run 505). The integrated profile collected from pulse 1 (blue) contains a single, sharp peak corresponding to the (111) Ag reflection. In data collected from subsequent pulses, the hot contribution is observed at lower diffraction angles due to thermal expansion of the lattice. The arrows indicate peaks from thermally expanded Ag which are observed at different positions during the run, suggestive of a continuously-evolving temperature gradient. The pressure of Ag in the cold pattern was determined to be 65.2 GPa based on the EoS of Sokolova *et al*.[1]. Ag has a calculated melting of temperature of 3800 K at this pressure[2], and the expected lattice parameter[1] at this temperature is indicated by the black dotted line on the figure, which appears to underestimate the volume of the expanded state. Data from this run are also shown in Supplementary Fig. 4 and Fig. 3. Source data are provided as a Source Data file.

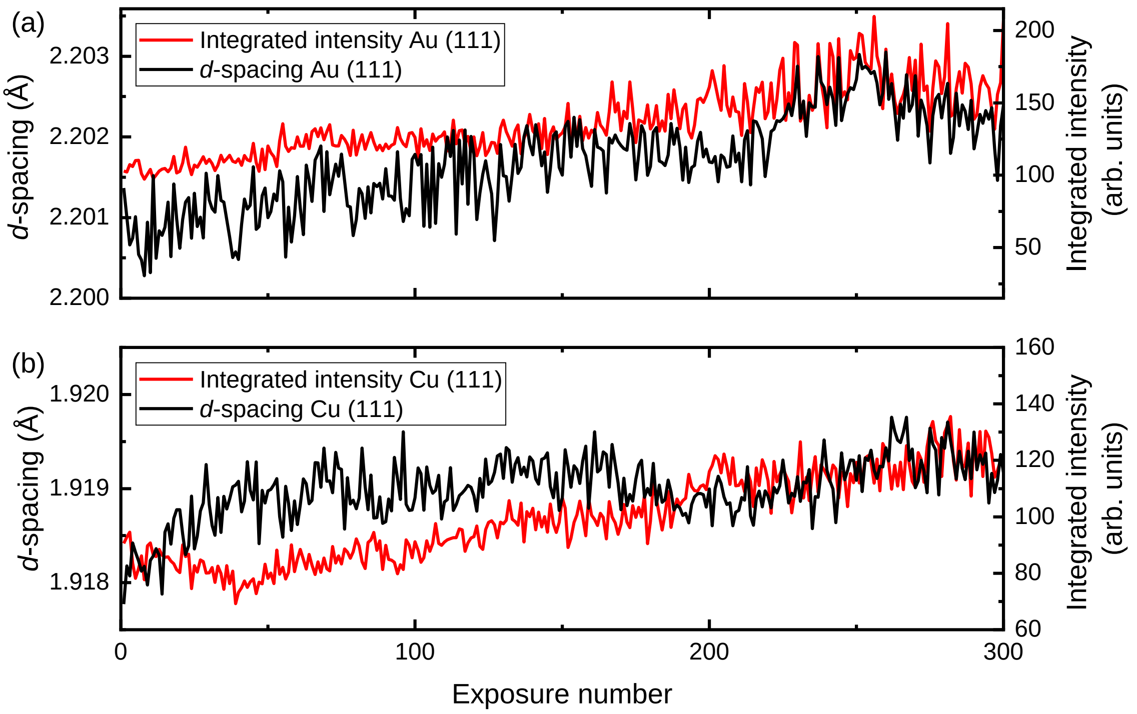

Supplementary Figure 9: Evolution of the integrated peak intensity and *d*-spacing for the most intense coupler reflections in runs collected from DAC 8. (a) shows the evolution of the (111) Au reflection in run 560, which was collected using 16% transmission (64.1 GPa), and (b) shows the evolution of the (111) Cu reflection in run 532, which was collected at 60% transmission (67.4 GPa). The data from these runs are also in Fig. S5. The observation of SI-bcc in these runs confirms that that H_2O is hot; however, the coupler reflections remain essentially unchanged during the run. Source data are provided as a Source Data file.

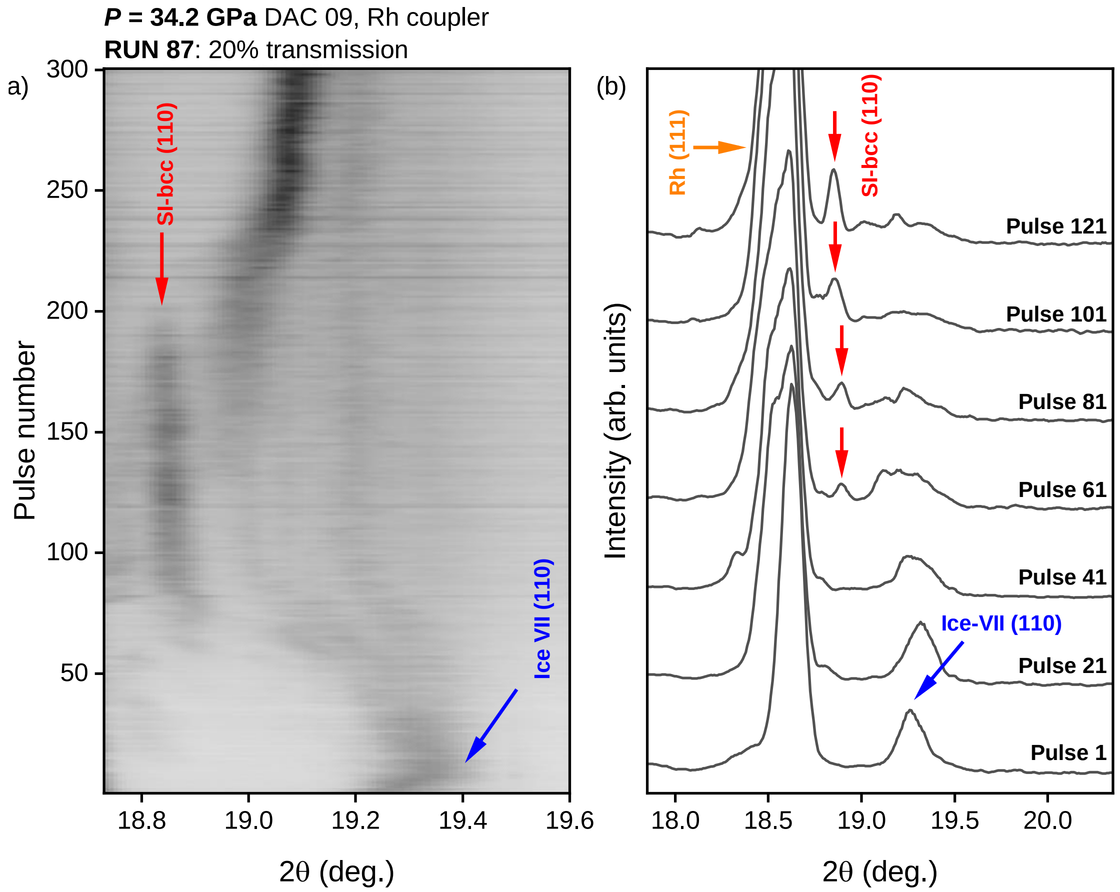

Supplementary Figure 10: XRD data collected at 34.2 GPa. Data were collected from the Rh coupler in DAC 9 using 20% X-ray transmission (run 87). The evolution of the integrated XRD patterns is shown in (a), and selected integration diffraction patterns are shown in (b). The intensity of the (110) ice VII reflection is significantly reduced after the appearance of the (110) SI-bcc reflection, suggesting that a large fraction of H_2O had transformed to the SI state. These data, which were collected using an X-ray focal spot size of 13 μm (FWHM) using a doughnut-type coupler with a 13-14 μm diameter hole size, illustrating that uniform heating of low Z samples using a high Z coupler is possible by careful choice of X-ray parameters and sample geometry. Uniform heating may also explain the observed shift of the ice VII reflection to higher angles at the start of the run, which is presumably due to thermal pressure, which is either not observed or much less pronounced in runs from other samples. Source data are provided as a Source Data file.

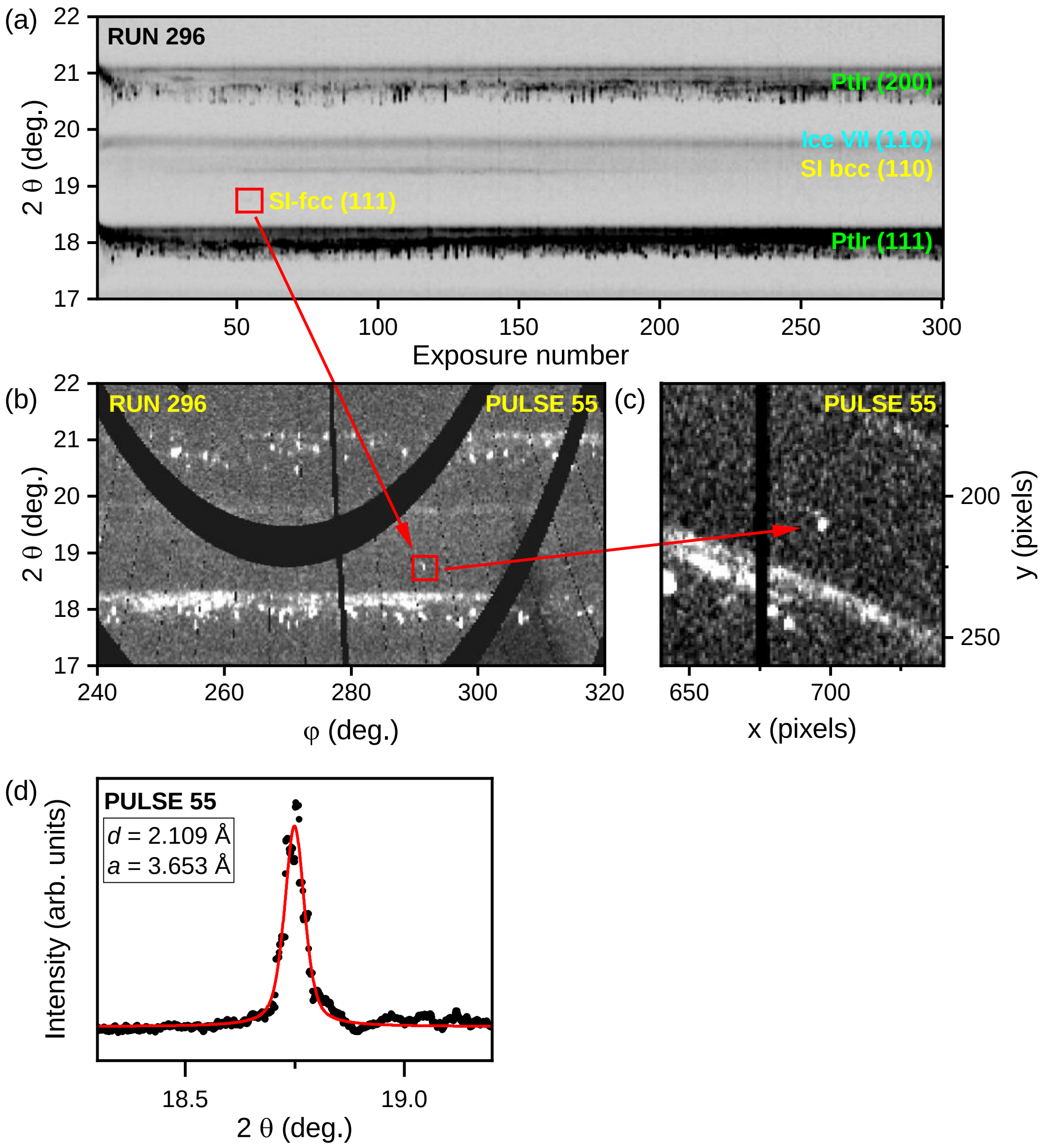

Supplementary Figure 11: Observation of SI-fcc in at 49.9 GPa. Data were collected from DAC 6 using a PtIr doughnut coupler and 15% X-ray transmission (run 296). (a) Intensity plot showing the changes in the integrated XRD signal during the run. The red rectangle highlights the region in which the SI-fcc (111) reflection was observed, which is too weak to be observed in the intensity plot, but is visible in (b) the unwrapped (2θ-φ) and (c) regular 2D XRD images. The integrated XRD pattern determined from an ROI is shown in panel (d), where the black points show the experimental data and the red line shows a fit using a Pseudo-Voigt function. The *d*-spacing and lattice parameter, *a*, calculated from the refined peak position fit are given in the inset. Source data are provided as a Source Data file.

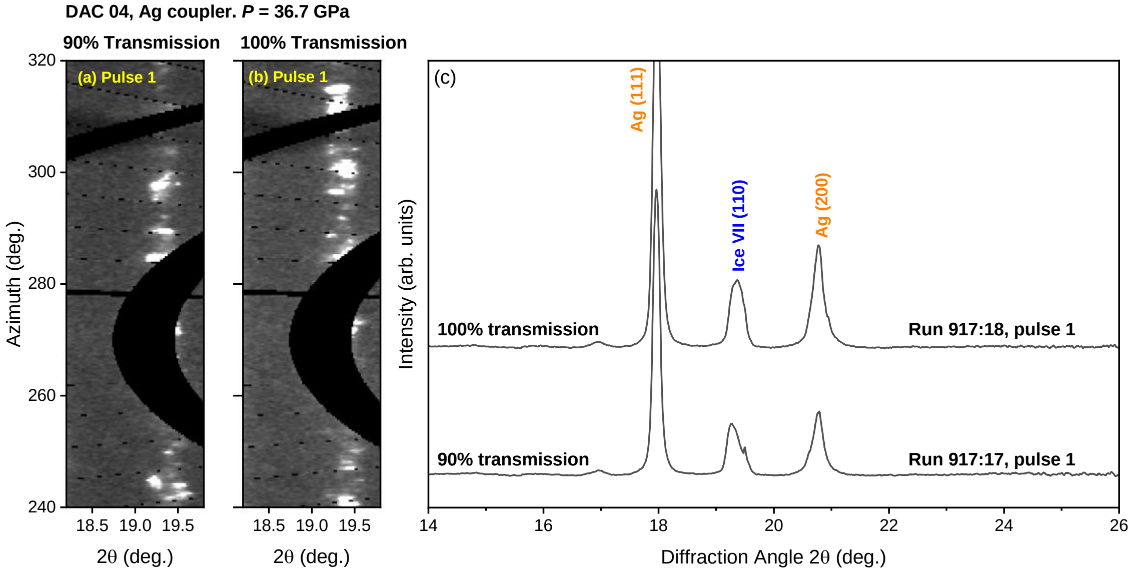

Supplementary Figure 12: XRD data collected during irradiation of the embedded Ag coupler in DAC 4 (36.6 GPa). (a) and (b) show the unwrapped (2θ-φ) XRD images collected from the first pulse of runs 917:17 (90% X-ray transmission) and 917:18 (100% X-ray transmission), respectively. These images illustrate the reappearance of the ice VII reflection following the heating cycle in run 917:17 (Fig. 6), and absence of contaminant peaks. The corresponding integrated patterns are shown in (c) to illustrated that that the XRD signal from the sample/coupler remain unchanged after heating. The small spike observed in the ice VII peak in the run 917:17 pattern originates from the diffracted signal which is partially blocked by one of the gaps in the AGIPD detector, and the weak peak at ~16.9° is from the Re gasket. Source data are provided as a Source Data file.

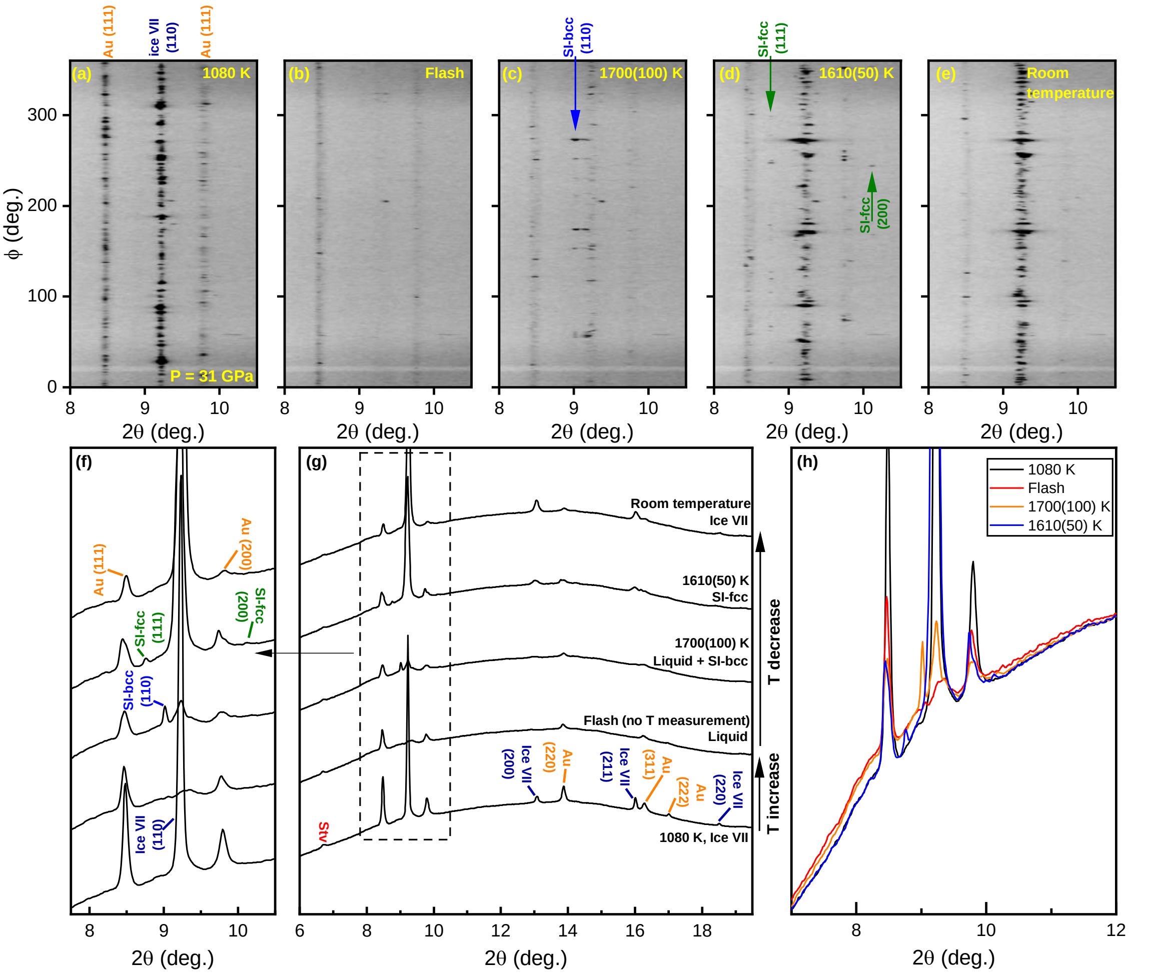

Supplementary Figure 13: XRD data collected during IR laser heating experiments. These experiments were performed using the same approach as Prakapenka *et al.*[3] (a)-(e) show the unwrapped (2θ-φ) XRD images and (f)-(h) show the corresponding integrated XRD patterns. The sample pressure was 31 GPa, and the sample was heated using an IR laser with Au as a laser absorber. (a) XRD from ice VII and the Au coupler are visible in XRD images collected from H_2O heated below its melting temperature. (b) Heating H_2O above its melting temperature results in the disappearance of the H_2O Bragg reflections and the appearance of diffuse scattering from the fluid phase. (c) On reduction of the laser power, molten ice is observed to coexist with SI-bcc. (d) Further decreasing the laser power results in the formation of SI-fcc, alongside the disappearance of the diffuse scattering signal disappears and an increase in the ice VII signal. (e) Diffraction from Au and ice VII are visible again on quench. In (g), Stv indicates a peak from stishovite, which was used as a thermal insulator. (h) illustrates the diffuse scattering signal in the flash and 1700(100) K patterns. Source data are provided as a Source Data file.

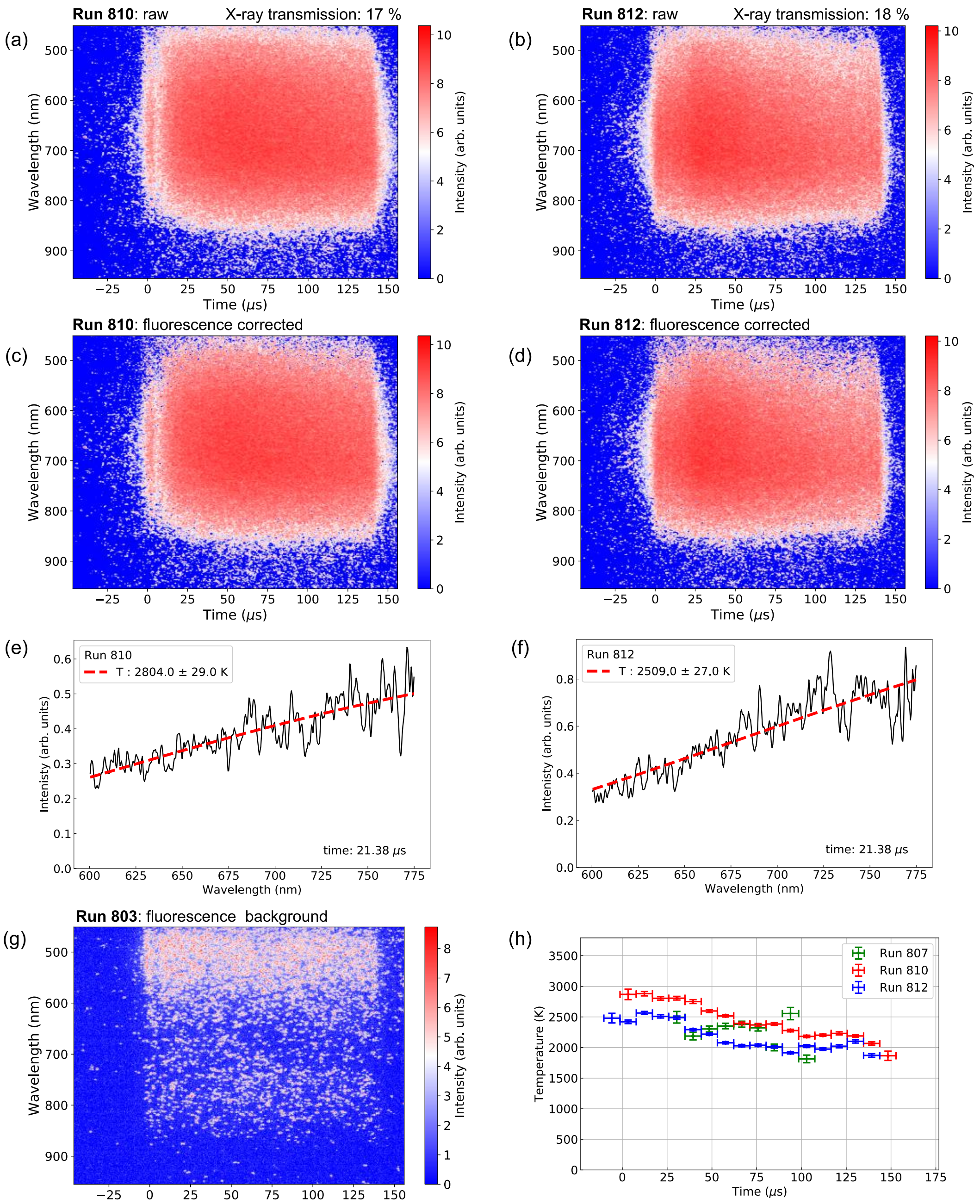

Supplementary Figure 14: Streaked optical pyrometry (SOP) data collected at 38.4 GPa. Data were collected during irradiation of the doughnut-type Au coupler in DAC 5. (a) and (b) show raw SOP spectrograms from runs 810 and 812, and (c) and (d) show the same images after subtraction of the fluorescence signal shown in (g), where time 0 corresponds to the start of the XFEL pulse train. Example Planck fits to thermal emission data in a 9.07 ns time window are shown for (e) run 810 and (f) run 812 at 21.38 μs, where the black solid lines show the experimental data and the red dashed line shows the fitted Planck curve. (g) Raw SOP spectrogram from run 803, which was collected at 7% X-ray transmission using the same coupler as runs 810 and 812. The observed optical emission originates from fluorescence from the diamond anvils and H_2O media, which results in strong signal in the 500-600 nm region which is not characteristic of thermal emission. This spectrogram was used as a fluorescence background which was subtracted from the thermal emission runs for this coupler. (h) Temperature as a function of time for runs 807, 810, and 812, which were collected with 13, 17, and 18% X-ray transmission, respectively. The horizontal error bars indicate the bin width and the vertical error bars correspond to one half of the standard deviation confidence from the Planck fit. Data points are only shown for spectra that provided reliable temperature determination according to the criteria outlined in reference [4]. Due to the low emissivity of H_2O in the temperature region probed here, SOP provides information on the surface temperature of the coupler, rather than providing a direct measurement of the temperature of the H_2O media. Source Data are provided as a Source Data file.

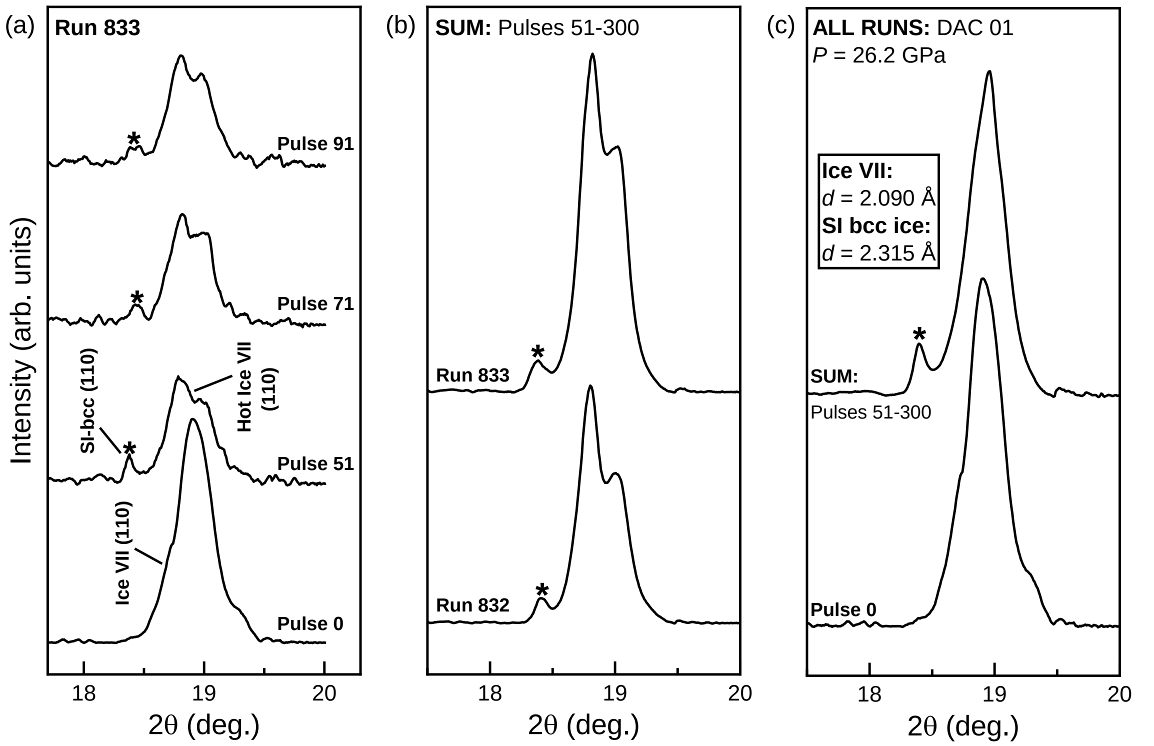

Supplementary Figure 15: **Integrated XRD data collected at 26.2 GPa.** Data were collected from the dispersed nanopower Fe_3O_4 coupler in DAC 1. Integrated XRD patterns from (a) individual XRD images collected during run 833 using 9% X-ray transmission, (b) XRD images summed over pulses 51-300 for runs 832 and 833 (7 and 9% transmission, respectively), and (c) XRD images from the first pulse (lower) and pulses 51-300 (upper) summed over all runs in which the (110) SI-bcc reflection was observed. The asterisk indicates the position of the (110) SI-bcc reflection. Heating results in a splitting of the (110) ice VII reflection due to the temperature gradient, which is clearly observed in the summed images from individual runs in (b). When data from all runs are summed, this results in a slight broadening of the peak. Source data are provided as a Source Data file.

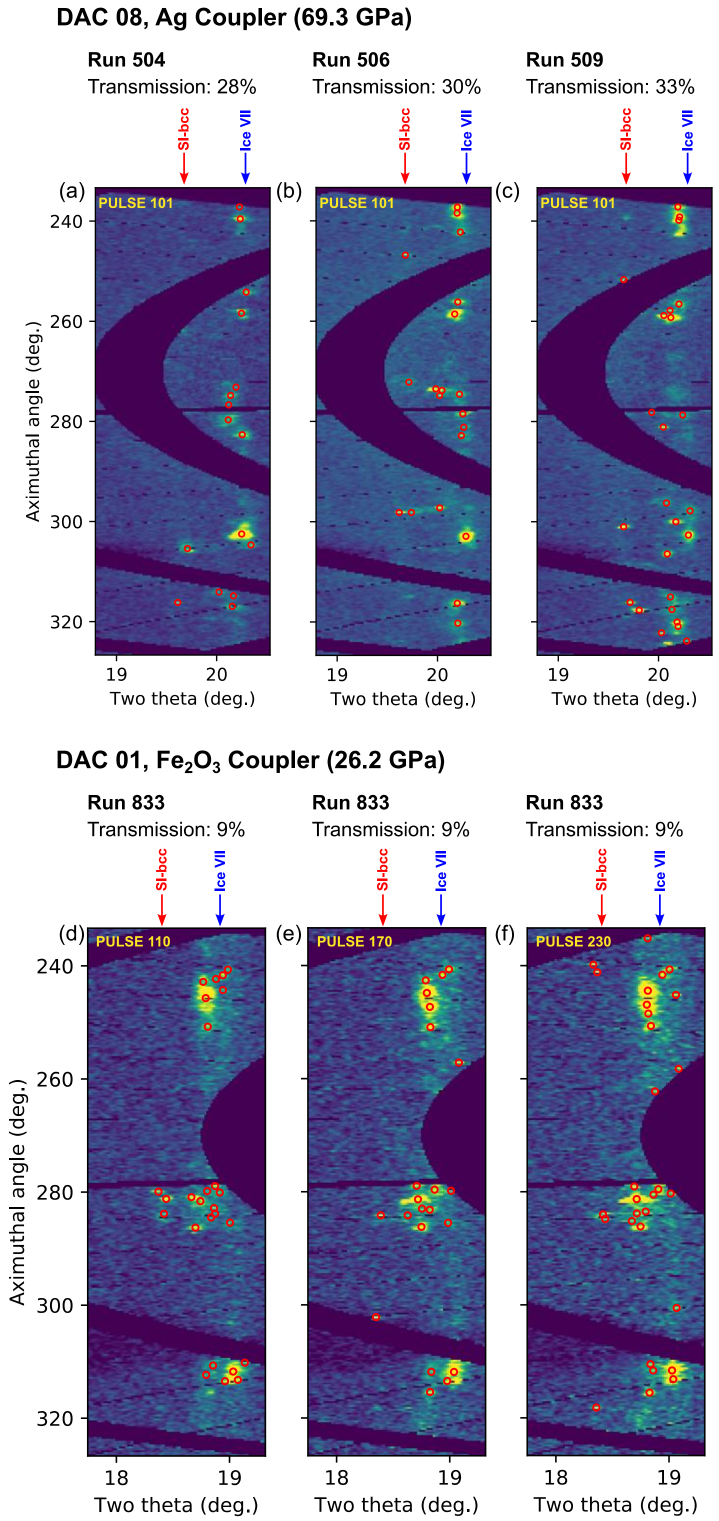

Supplementary Figure 16: **Results of the peak finding algorithm for a selection of unwrapped (2θ-φ), single pulse AGIPD images.** (a), (b), and (c) show the results for XRD images collected from the 101th pulse XFEL pulse in runs 504, 505, and 509, which were collected at 28%, 30%, and 33% X-ray transmission, respectively, from the Ag coupler in DAC 8 (69.3 GPa). (d), (f), and (f) show the results for XRD images collected from pulses 110, 170, and 230 in run 883, which was collected from the nano-dispersed Fe_3O_4 coupler in DAC 1 (26.2 GPa). The red circles indicate the position of individual diffraction spots found by the spot finding algorithm. The arrows indicate the peak positions of the (110) ice VII reflection in the cold pattern (blue), and the (110) SI-bcc reflection in the hot pattern (red). Source data are provided as a Source Data file.

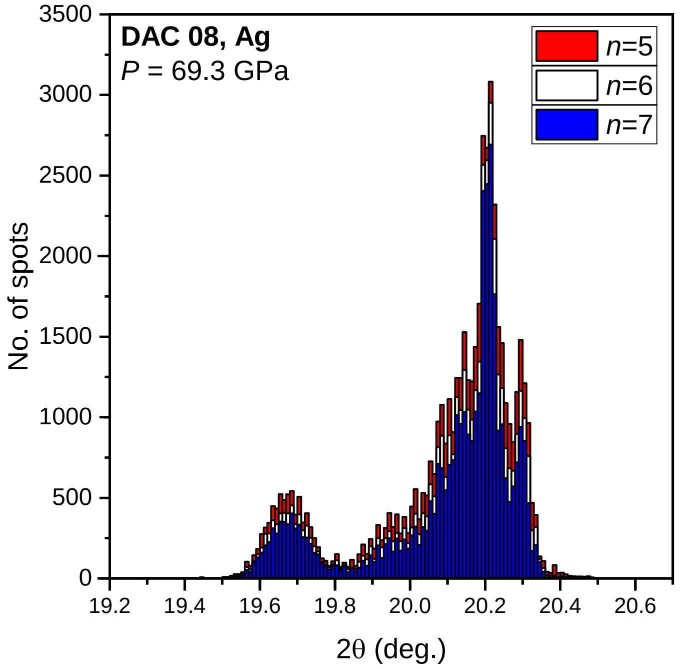

Supplementary Figure 17: Histogram showing the results of the spot finding algorithm for different threshold values. Histograms were compiled from runs collected from the Ag doughnut coupler in DAC 8 (69.3 GPa). Histograms were created using XRD images from pulses 51-300 in runs where SI-bcc was observed. The plot shows histograms for different values of *n*, which is used to define the threshold via $\bar{\iota}_{bk} + n\sigma_{i_{bk}}$. Although the choice of *n* influences the number of spots located by the algorithm, it does not influence the results. The value *n* = 6 was chosen for data analysis based on visual inspection of peaks located in a small subset of images (see Supplementary Fig. 16). Larger values of *n* resulted in peaks being missed by the algorithm, and smaller values tended to result in peaks incorrectly being identified in the noise. Source data are provided as a Source Data file.

Supplementary References

1. Sokolova, T. S., Dorogokupets, P. I., Dymshits, A. M., Danilov, B. S. & Litasov, K. D. Microsoft excel spreadsheets for calculation of P–V–T relations and thermodynamic properties from equations of state of MgO, diamond and nine metals as pressure markers in high-pressure and high-temperature experiments. *Comput. Geosci.* **94**, 162–169 (2016).
2. Hieu, H. K. & Ha, N. N. High pressure melting curves of silver, gold and copper. *AIP Adv.* **3**, 112125 (2013).
3. Prakapenka, V. B., Holtgrewe, N., Lobanov, S. S. & Goncharov, A. F. Structure and properties of two superionic ice phases. *Nat. Phys.* **17**, 1233–1238 (2021).
4. Ball, O. B. *et al.* Dynamic optical spectroscopy and pyrometry of static targets under optical and x-ray laser heating at the European XFEL. *J. Appl. Phys.* **134**, 055901 (2023).
