# Supplementary material for: Phase transition kinetics of superionic H2O ice phases revealed by Megahertz X-ray free-electron laser-heating experiments
Source: Nat Commun. 2024 Sep 23;15:8256. doi: 10.1038/s41467-024-52505-0 (PMC11420352; doi:10.1038/s41467-024-52505-0)
Supplement: Supplementary file 3 — Description of Additional Supplementary Files [file 41467_2024_52505_MOESM3_ESM.pdf]

Description of Additional Supplementary Files

Supplementary Movie 1: Unwrapped (2θ-ϕ) 2D diffraction images (left) and the corresponding histogram (right) from run 505, which was collected during irradiation of the Ag doughnut-type coupler in DAC 8 (69.3 GPa) with 300 pulses at 29% X-ray transmission. The red circles indicate the diffraction spots located by the spot finding algorithm (note: the diameters of the circles are unrelated to individual spot sizes). The histogram was constructed from images collected from pulse 51 onwards, after the system reached a balance between X-ray heating and heat loss via thermal conduction. The width of each bin is 0.01 degrees. The resultant histogram is shown in Fig. 4(a).

Supplementary Movie 2: Unwrapped (2θ-ϕ) 2D diffraction images (left) and the corresponding histogram (right) from run 917:17, which was collected during the irradiation of the Ag doughnut-type coupler in DAC 4 (36.7 GPa) with 300 pulses at 90% X-ray transmission. The red circles indicate the diffraction spots located by the spot finding algorithm (note: the diameters of the circles are unrelated to individual spot sizes). The histogram was constructed from images collected from pulse 51 onwards, after the system reached a balance between X-ray heating and heat loss via thermal conduction. The width of each bin is 0.01 degrees. The intensity of the diffracted signal from both SI-bcc and ice VII reduced as the run progressed, and individual spots are barely visible in the XRD image collected from pulse 30. The significant reduction in the intensity of the ice VII/SI-bcc diffracted signal is attributed to melting, which is supported by the strong ice VII signal and absence of chemical reaction products in the first pulse pattern of the subsequent run. No evidence of the SI fcc (111) reflection was observed, which would be expected to be present at approximately 18.4°. Selected unwrapped (2θ-ϕ) 2D diffraction images from this run are shown in Fig. 6.
